# Supplementary material for: Morning Engagement of Hepatic Insulin Receptors Improves Afternoon Hepatic Glucose Disposal and Storage
Source: bioRxiv. 2024 Sep 27:2024.09.25.614969. Preprint. [Version 1] doi: 10.1101/2024.09.25.614969 (PMC11463395; doi:10.1101/2024.09.25.614969)

SUPPLEMENTAL MATERIALS

Morning Engagement of Hepatic Insulin Receptors Improves Afternoon Hepatic Glucose Disposal and Storage

Hannah L. Waterman[1], Mary Courtney Moore[1], Marta S. Smith[1], Ben Farmer[1], Kalisha Yankey[1], Melanie Scott[1], Dale S. Edgerton[1], Alan D. Cherrington[1]

[1]Department of Molecular Physiology & Biophysics, Vanderbilt University School of Medicine

Western Blotting

The following primary antibodies were used in western blotting analysis: pAkt (#9271, Cell Signaling Technology, Danvers, MA), tAkt (#9272, Cell Signaling Technology), pGS (#98348, Cell Signaling Technology), tGS (#3893, Cell Signaling Technology), pGSK3β (#9336, Cell Signaling Technology), tGSK3β (#9315, Cell Signaling Technology), and GK (#sc-17819, Santa Cruz Biotechnology). Anti-mouse and Anti-rabbit IgG (H+L), HRP conjugate secondary antibodies were purchased from Promega (Madison, WI).

Protein samples were loaded in order by group for all western blot images. The manuscript expresses these data as phosphorylated proteins relative to total protein for Akt, GS, and GP. GK is expressed over Cyclophilin B. The image for each respective protein can be viewed below.

AM Po Ins (No PM Clamp and PM Clamp) Western Blot Images

Phosphorylated Akt (pAkt)

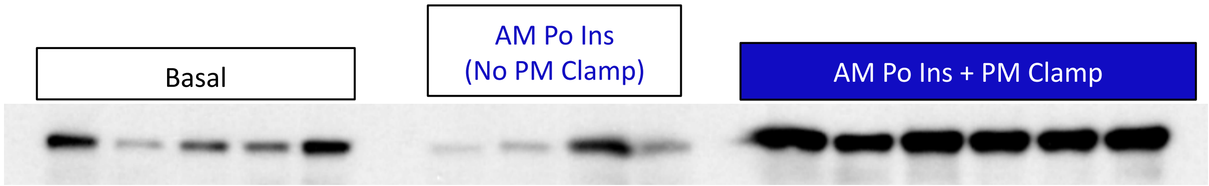

Total Akt (tAkt)

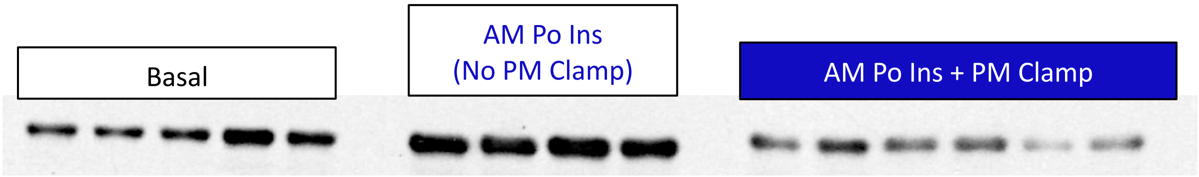

GK

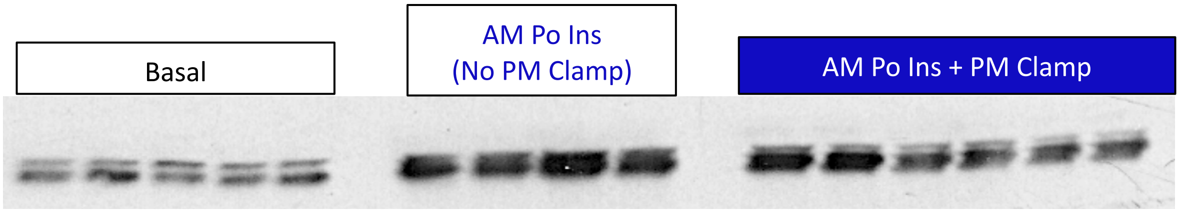

Cyclophilin B

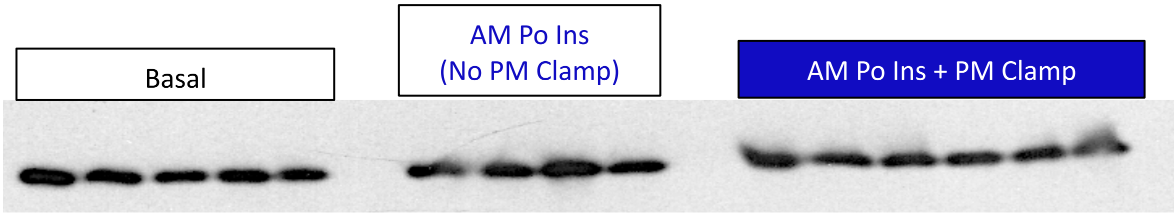

Phosphorylated GS (pGS)

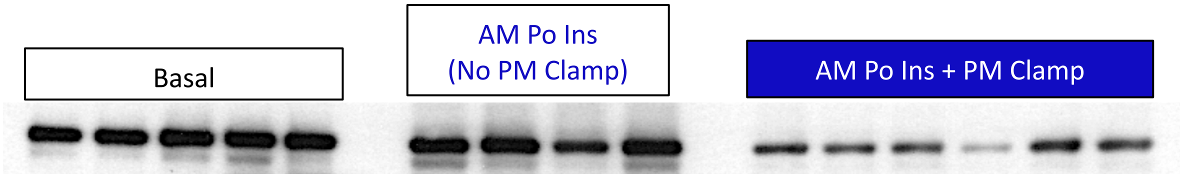

Total GS (tGS)

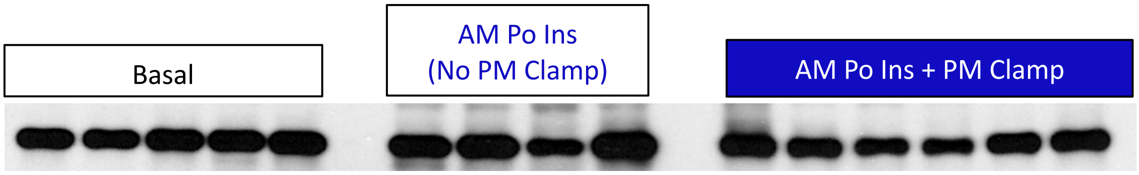

Phosphorylated GP (pGP)

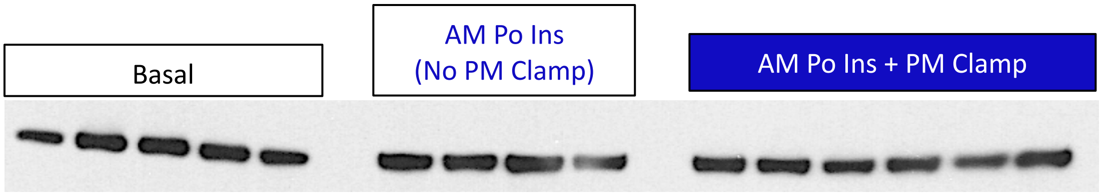

Total GP (tGP)

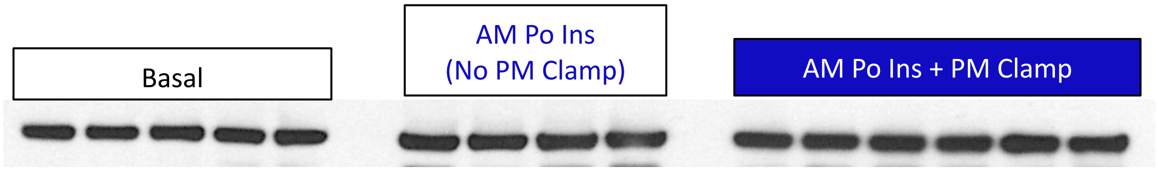

AM Pe Ins (No PM Clamp and PM Clamp) Western Blot Images

Phosphorylated Akt (pAkt)

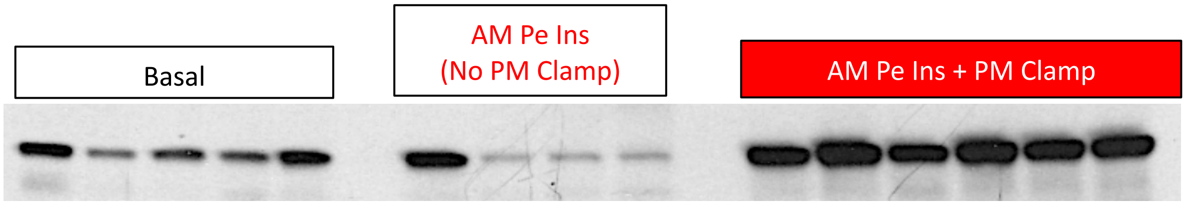

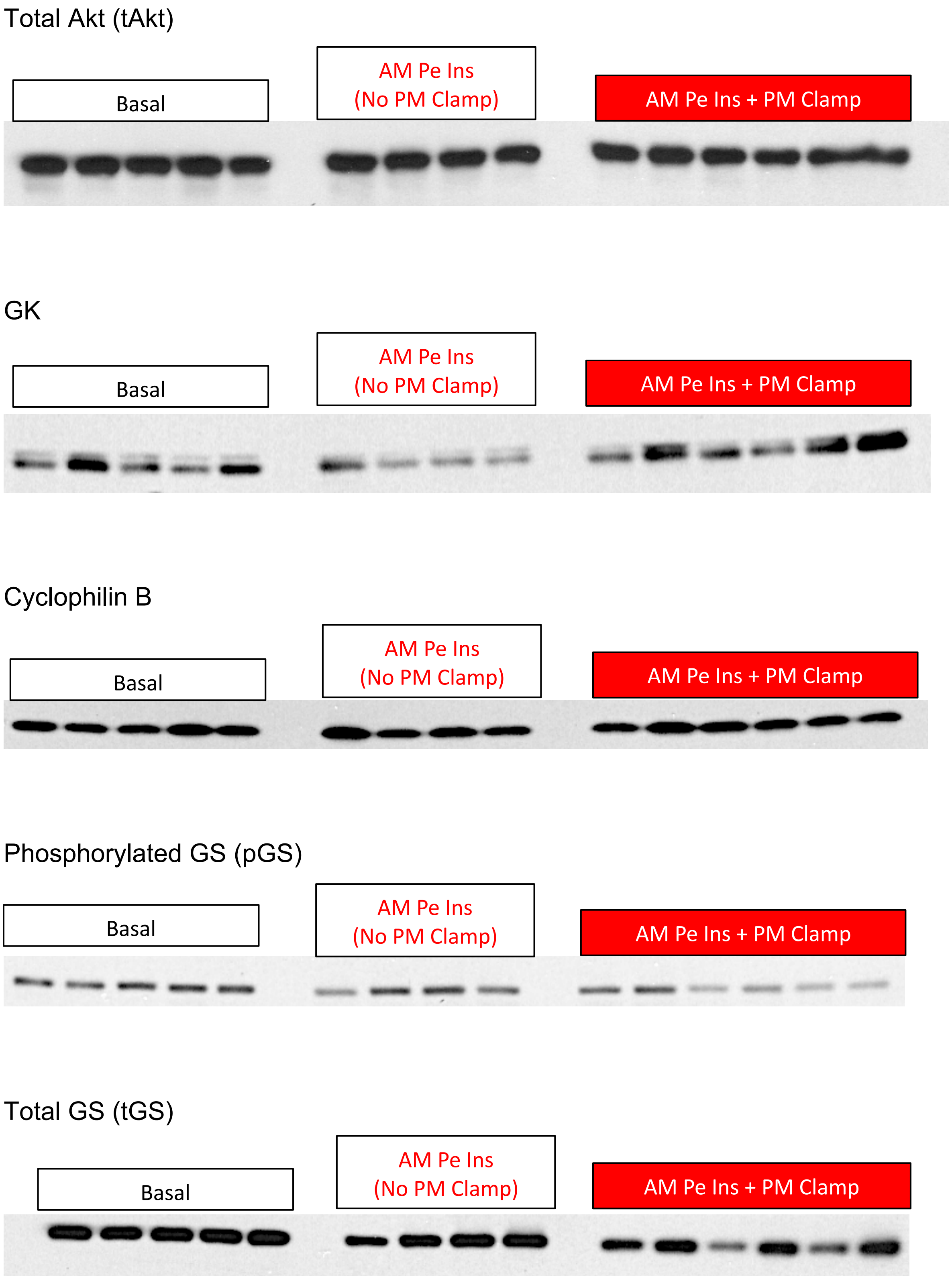
Total Akt (tAkt)
Basal
AM Pe Ins
(No PM Clamp)
AM Pe Ins + PM Clamp
GK
Basal
AM Pe Ins
(No PM Clamp)
AM Pe Ins + PM Clamp
Cyclophilin B
Basal
AM Pe Ins
(No PM Clamp)
AM Pe Ins + PM Clamp
Phosphorylated GS (pGS)
Basal
AM Pe Ins
(No PM Clamp)
AM Pe Ins + PM Clamp
Total GS (tGS)
Basal
AM Pe Ins
(No PM Clamp)
AM Pe Ins + PM Clamp

Phosphorylated GP (pGP)

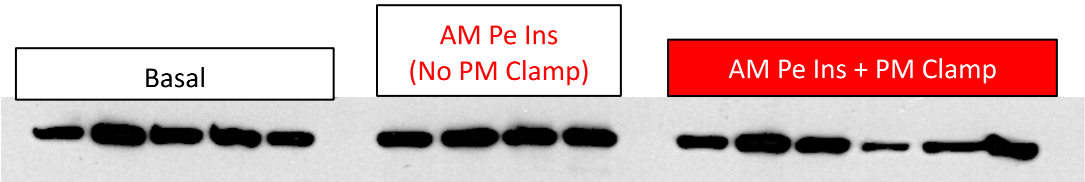

Total GP (tGP)

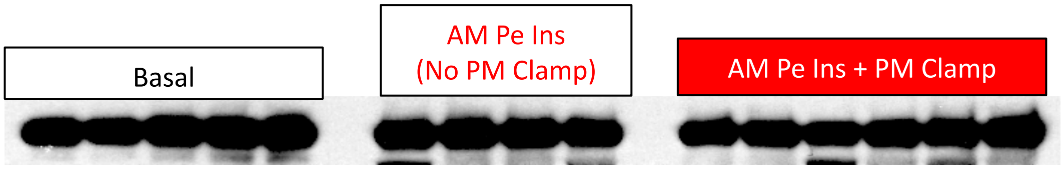

Supplement: Supplement 1 [file media-1.pdf]
